# A third mitochondrial RNA polymerase in the moss *Physcomitrella patens*

**DOI:** 10.1007/s00294-013-0405-y

**Published:** 2013-09-12

**Authors:** Uwe Richter, Björn Richter, Andreas Weihe, Thomas Börner

**Affiliations:** 1 Institut für Biologie-Genetik, Humboldt-Universität zu Berlin, Chausseestr. 117, 10115 Berlin, Germany; 2 Present Address: Research Programs Unit, Molecular Neurology, University of Helsinki, Haartmaninkatu 8, 00290 Helsinki, Finland

**Keywords:** Chloroplast transcription, Mitochondrial transcription, NEP, PEP, Phage-type RNA polymerase, *Physcomitrella*

## Abstract

In most organisms, the mitochondrial genes are transcribed by RNA polymerases related to the single-subunit RNA polymerases of bacteriophages like T3 and T7. In flowering plants, duplication(s) of the *RpoTm* gene coding for the mitochondrial RNA polymerase (RPOTm) led to the evolution of additional RNA polymerases transcribing genes in plastids (RPOTp) or in both mitochondria and plastids (RPOTmp). Two putative RPOTmp enzymes were previously described to be encoded by the nuclear genes *RpoTmp1* and *RpoTmp2* in the moss *Physcomitrella patens*. Here, we report on a third *Physcomitrella* *RpoT* gene. We determined the sequence of the cDNA. Comparison of the deduced amino acid sequence with sequences of plant organellar RNA polymerases suggests that this gene encodes a functional phage-type RNA polymerase. The 78 N-terminal amino acids of the putative RNA polymerase were fused to GFP and found to target the fusion protein exclusively to mitochondria in *Arabidopsis* protoplasts. *P. patens* is the only known organism to possess three mitochondrial RNA polymerases.

**Electronic supplementary material:**

The online version of this article (doi:10.1007/s00294-013-0405-y) contains supplementary material, which is available to authorized users.

## Introduction

Mitochondrial transcription is strictly dependent on nuclear gene-encoded phage-type RNA polymerases since all eukaryotes, with the exception of the jacobid protists, seem to lack a mitochondrial bacterial-type RNA polymerase (Burger et al. 2013). On the contrary, chloroplasts have retained a eubacterial RNA polymerase with chloroplast gene-encoded core subunits and nuclear gene-encoded sigma factor(s) from their cyanobacterial ancestor. In angiosperms, the bacterial-type RNA polymerase (called PEP for *p*lastid-*e*ncoded *p*lastid RNA *p*olymerase) is not sufficient for transcription of the chloroplast genes. The activity of one or two phage-type RNA polymerase(s) (called NEP for *n*uclear-*e*ncoded *p*lastid RNA *p*olymerase) has to complement PEP activity. NEP has evolved in angiosperms by duplication(s) of the *RpoTm* gene for the mitochondrial RNA polymerase (*RpoT* for *R*NA *po*lymerase of phage *T*3/7-type; *m* for mitochondrial (Liere et al. 2011). *RpoT* genes encoding mitochondrial and/or plastid phage-type polymerases have been identified in several land plants. Eudicotyledonous plants such as *Arabidopsis* and *Nicotiana* posses at least three *RpoT* genes coding for a mitochondrial (RPOTm), a plastid (RPOTp) and a dual-targeted polymerase (RPOTmp) (Hedtke et al. 2000, 2002; Kobayashi et al. 2002, a, b). Nuclear genomes of monocots investigated thus far harbour not more than two *RpoT* genes, one coding for a mitochondrial RNA polymerase (RPOTm) and the other for a plastidial enzyme (RPOTp) (Chang et al. 1999; Emanuel et al. 2004; Ikeda and Gray 1999), seemingly lacking the *RpoTmp* gene encoding the dual-targeted RPOT polymerase found in eudicots. This *RpoTmp* originated most likely after *RpoTp* from a second duplication of the *RpoTm* gene during angiosperm evolution (Liere et al. 2011). In the basal angiosperm *Nuphar advena*, two duplications of the *RpoTm* gene gave rise to the plastid enzyme RPOTp and to a second mitochondrial RNA polymerase (Yin et al. 2010).

While *RpoT* genes from angiosperms have been well characterised [reviewed in (Liere et al. 2011)], almost nothing is known about the evolution of those genes in other lineages of the plant kingdom. Only one *RpoT* gene coding for the mitochondrial RNA polymerase was detected in the lycophyte *Selaginella moellendorffii* (Yin et al. 2009). In the deeper branching leafy moss *Physcomitrella patens*, however, two nuclear *RpoT* genes were reported to exist (Kabeya et al. 2002; Richter et al. 2002). Dual targeting of the two *RpoT* gene products to both mitochondria and plastids being the result of two different translational starts has been described, and the polymerases were designated as PpRPOTmp1 and PpRPOTmp2 (Richter et al. 2002). For both PpRPOTmp1 and PpRPOTmp2, translation initiation at the first of two in-frame AUG start codons was found to compose a polypeptide with N-terminal plastidial targeting properties, whereas initiation at the downstream AUG gave rise to a mitochondrial protein (Richter et al. 2002). More recently, Kabeya and Sato (2005) demonstrated that translation initiation from the first AUG at the *RpoTmp1* and *RpoTmp2* transcripts was repressed for all constructs with the native 5′UTR present. Thus, targeting to mitochondria or chloroplasts of these enzymes might be tightly regulated by alternative usage of the two start codons.

Phylogenetic analyses show a distinct group of the plant *RpoT* sequences clearly separated from those of fungi and animals. In concordance with this finding, the plant specific gene structure is highly conserved in all plant *RpoT* genes investigated thus far (Richter et al. 2002). Within this group, the RPOT polymerases of *Physcomitrella patens* are separated from the angiosperm polymerases, with the latter falling into two groups with plastid-localised enzymes on one hand, and mitochondrial- and dual-targeted polymerases on the other (Liere and Börner 2007; Richter et al. 2002). The duplication event that led to the *RpoT* gene copies in *P. patens*, thus, must be considered to have occurred independently from the one giving rise to the *RpoT* gene family in angiosperms. Furthermore, it shows that the duplication giving rise to a group of exclusively plastid-localised RPOTp gene products of angiosperms is predating the duplication event that led to the appearance of the third gene as found in eudicot plants, such as *A. thaliana* or *N. sylvestris* (Richter et al. 2002).

The moss *P. patens* (Hedw.) Bruch and Schimp is a widely spread species which colonizes open habitats in cold temperate zones. The occurrence of homologous recombination in its nuclear genome and several other aspects of its biology, e.g., small size, short life cycle, a relatively easy cultivation procedure, its basal phylogenetic position, an extensive EST database and the fact that it is predominantly haploid make *Physcomitrella* ideal for molecular and comparative studies (Cove 2005). Moreover, the complete genome sequence has been published (Rensing et al. 2008).

Surprisingly, BLAST analysis of the genome sequence of *P. patens* revealed a third genomic locus (provisionally designated as *PpRpoT3*) bearing close similarity to plant phage-type RNA polymerases. We have determined the sequence of this third *Physcomitrella* *RpoT* gene and of its transcript. We report here that it encodes an RNA polymerase that is targeted to the mitochondria.

## Materials and methods

### *PpRpoT3* cDNA cloning

The BLAST analysis of the published *Physcomitrella* whole genome sequence (Rensing et al. 2008) revealed a third genomic locus potentially encoding a phage-type RNA polymerase on PHYPAscaffold_241 (NW_001865497). Putative coding regions were used to generate primers for the amplification of cDNA ends. 3′- and 5′-RACE reactions employed the CapFishing Full-length cDNA Premix kit (Seegene; Eschborn, Germany) and Phusion hot start DNA polymerase (New England Biolabs GmbH, Frankfurt am Main, Germany) according to the protocols of the manufacturers. For cDNA synthesis, 1 μg of polyA-enriched *Physcomitrella* protonema RNA was reverse transcribed using SuperScript™ III RNase H-reverse transcriptase kit (Life Technologies, Darmstadt, Germany). Notably, 5′RACE reverse transcription employed two gene-specific primers annealing to the in silico-predicted lower exon 1 region (5racePpT3gsp1/5racePpT3gsp2). While 3′- and 5′-RACE adaptor primers were provided in the kit, gene-specific primers (gsp) were employed for two rounds of PCR to enhance the specificity of the reaction (see Supplementary Table 1). To verify the cloned transcript ends, a primer walking strategy was used until no specific amplification products were obtained after a second round of 35 PCR cycles (5racePpT3gsp7 and 3racePpT3gsp4). After the identification of both *PpRpoT3* transcript termini, the whole *PpRpoT3* coding region (the complete cDNA sequence was deposited in the EMBL database under accession number Hx2000029983) was amplified using primers PpT3fw1 and PpT3m3169, and the fragment was cloned into pDrive (pDriveRpoT3cDNA). To map the *PpRpoT3* gene structure, subsequent sequencing of the whole *PpRpoT3* cDNA employed primers used in previous steps of the cloning procedure. Sequencing primers are shown in Supplementary Table 1.

### Generation of PpRPOT3-GFP fusion constructs

To generate the pPpRPOT3-GFP constructs and to drive the expression of the fusion protein PpRPOT3-GFP, a *PpRpoT3* fragment containing the full 5′UTR, and encoding 78 N-terminal amino acids of PpRPOT3 was amplified from pDriveRpoT3cDNA using primers PpT3gfpfwXbaI (cactctagaCTGCTTGCGTTGCTTTGC) and PpT3gfprevSalI (atagtcgacCAAGGATGTCTTCCAGAGGTG; lowercase letters correspond to non-annealing nucleotides for the introduction of restriction sites). PpT3gfpXba1-2 (cactctagaGAGTTGAATACTATGTGGC) was used for the amplification of a construct lacking all, but 12 nt of the 5′UTR. PCR products were *Xba*I/*Sal*I-digested and ligated into the *Spe*I/*Sal*I-cleaved vector pOL−GFP S65C (Peeters et al. 2000). Mitochondrial CoxIV-GFP and plastidial RecA-GFP (Peeters et al. 2000) control constructs were kindly provided by Ian Small (UWA, Perth, Australia).

### Transient expression in Arabidopsis protoplasts and microscopy

Protoplasts were isolated from *Arabidopsis* *thaliana* leaves grown for 4 weeks under long day conditions (23 °C, 16/8 h light/dark) as described before (Yoo et al. 2007). Briefly, 100 μl protoplasts (2 × 10^6^ protoplasts/ml) were transfected with 20 μg pOL-PpRpoT3gfp plasmid in 40 % polyethylene glycol 4000, 0.8 M mannitol, and 1 mM CaCl_2_. Transformed protoplasts were kept in the dark and examined 48 h after transfection by confocal laser scanning microscopy with a Leica TCS SP2 using 488-nm excitation and two-channel measurement of emission from 510 to 580 nm (green/GFP) and >590 nm (red/chlorophyll).

### Phylogenetic analyses

Sequence information was retrieved from the National Center for Biotechnology Information (http://www.ncbi.nlm.nih.gov/BLAST) employing the BLASTP and TBLASTN algorithms and from the Joint Genome Institute (http://genome.jgi-psf.org/). For translation and alignment, sequences were subsequently imported into GENEIOUS (Drummond et al. 2010). Multiple protein sequence alignments were generated using CLUSTALW (Thompson et al. 1994) implemented in the GENEIOUS package. The phylogeny was reconstructed by the PhyML algorithm V3.0 (Guindon et al. 2010) with 100 bootstrap replicates. The aligned sequence data comprised 894 amino acids lacking only the less conserved N-terminal portion of the proteins. For the reconstruction, the LG amino acid replacement model was employed. A discrete gamma distribution with four categories was assumed to approximate the continuous function (shape: 1,004/inv: 0,138). Essentially, the same tree topology was obtained employing Bayesian (Ronquist and Huelsenbeck 2003) and neighbor joining analysis (Saitou and Nei 1987). The tree was rooted with the four green algae RPOT proteins indicated. Tree reconstruction was based on a multiple alignment of 54 plant RPOT sequences (Supplementary Table 2).

## Results and discussion

### Characterization of the third gene encoding a phage-type RNA polymerase in *Physcomitrella*

**Fig. 1:**
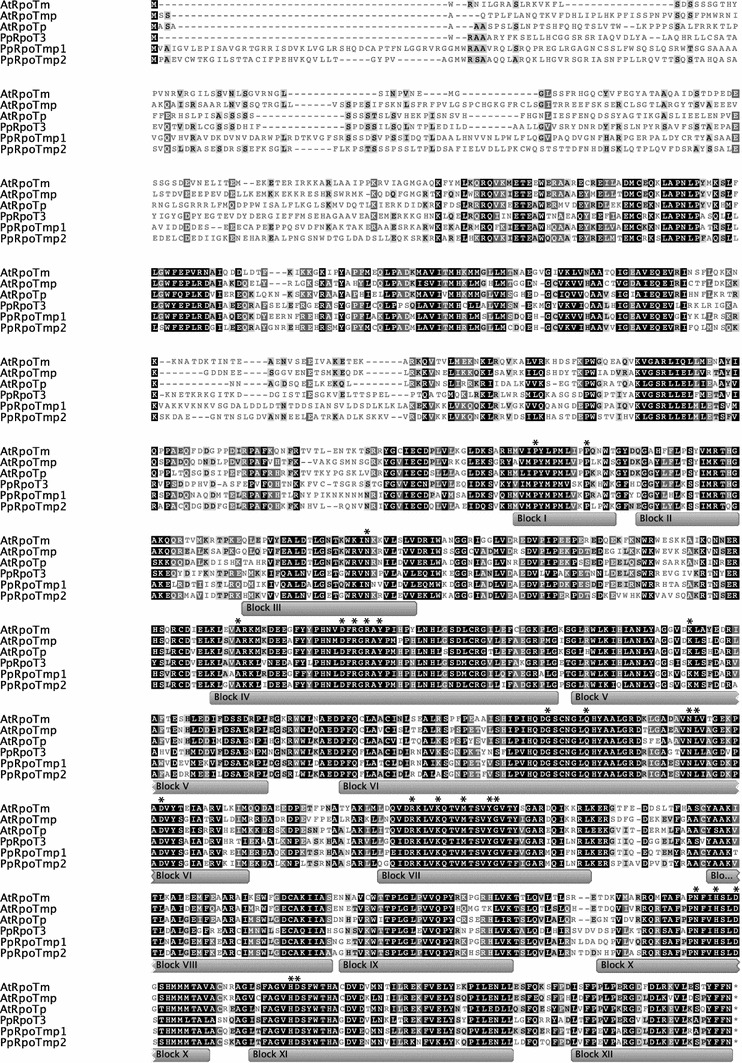
Amino acid sequence alignment. Amino acid sequences were compared among *Arabidopsis* and *Physcomitrella* RPOT polymerases using the ClustalW algorithm. Sequences with accession numbers: AtRPOTm (CAA69331), AtRPOTp (CAA70210), AtRPOTmp (CAC17120); PpRPOTmp1 (CAC95163); PpRPOTmp2 (CAC95164). *Grey boxes* indicate conserved *blocks* in the RPOT polymerase family; functionally crucial residues (McAllister and Raskin 1993; Sousa et al. 1993) are indicated by *asterisks*

**Fig. 2:**
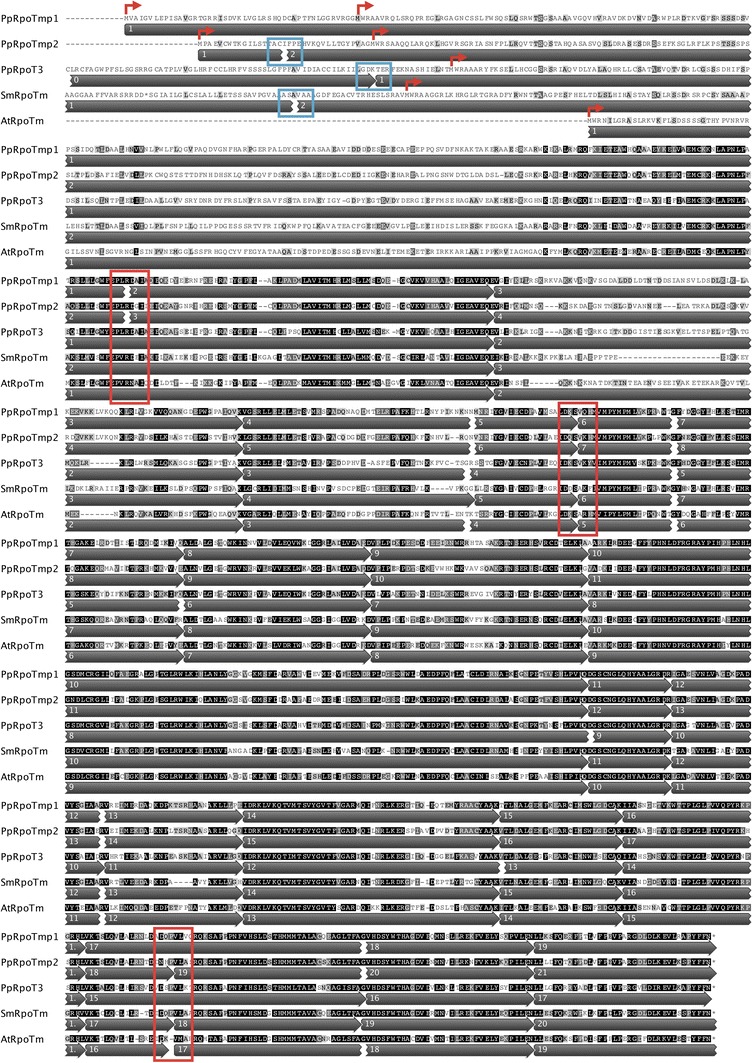
ClustalW-derived protein alignment. All three RPOT proteins from *Physcomitrella* were aligned with SmRPOTm of *Selaginella moellendorffii* and AtRPOTm of *Arabidopsis thaliana* using the ClustalW algorithm. *Grey arrows* below the sequence indicate exons and numbers inside indicate exon numbers. While *red arrows* mark putative translational start sites, *blue boxes* highlight intronic insertions in the putative 5′UTRs. *Red boxes* mark intron positions found in at least one of the *Physcomitrella RpoT* genes, but lacking in *PpRpoT3*

To identify the precise position and length of the open reading frame encoding the third phage-type RNA polymerase, we determined the full-length cDNA sequence. Transcripts of the gene were present in RNA preparations from protonema tissue, i.e., the *RpoT3* gene is active. The full-length cDNA comprising 3,496 bp was obtained by aligning fragments produced by RT–PCR and RACE. It constitutes 235 bp of untranslated leader, the protein coding sequence of 3,030 bp, and a 3′-untranslated sequence of 231 bp. Therefore, the predicted PpRPOT3 protein comprises 1,010 amino acids and it shows 46–48 % identity with the three *Arabidopsis* RPOTs. While the two *Physcomitrella* RPOT polypeptides RPOTmp1 and RPOTmp2 share 59 % identity, the scores for RPOT3 are significantly lower; 53 % with RPOTmp1 and 51 % with RPOTmp2. Interestingly, the 46–48 % identity scores of RPOT3 with the three *Arabidopsis* RPOTs are higher then those of RPOTmp1 (43–47 %) and RPOTmp2 (43–46 %). Figure 1 shows an alignment of the *Arabidopsis* and *Physcomitrella* RPOT polymerases demonstrating the high degree of similarity and the conservation of functionally essential residues and motifs in PpRPOT3. Thus, the derived amino acid sequence is consistent with *PpRpoT3* encoding a functional RNA polymerase. To elucidate the gene structure of the third *Physcomitrella RpoT* gene, the cDNA was aligned with the genomic sequence taken from the JGI genome browser (http://genome.jgi-psf.org/). The two published *RpoT* genes *RpoTmp1* (7.3 kb) and *RpoTmp2* (8.7 kb) contain 18 and 20 introns, respectively (Richter et al. 2002). In *PpRpoT3* (7.3 kb), only 17 introns are found. Compared with the *RpoT* gene structure of flowering plants, *PpRpoT3* contains one additional intron in the 5′ non-coding part of the gene, intron 4 and 16 (as of *Arabidopsis RpoTm*) are missing in *PpRpoT3* (Fig. 2).

The insertion sites of all other common introns are precisely conserved relative to the aligned amino acids sequences between *PpRpoT3*, the two other *Physcomitrella* genes and those of *Arabidopsis* (Fig. 2).

### Phylogenetic analysis

**Fig. 3:**
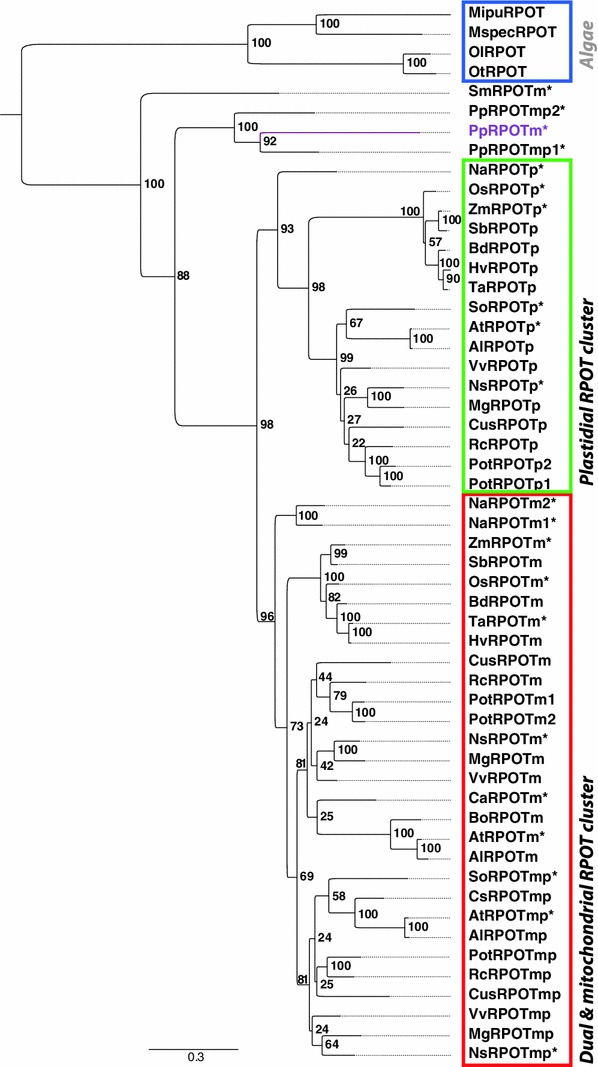
Phylogenetic analysis of plant RPOT proteins. The phylogeny was reconstructed by the PhyML algorithm V3.0 (Guindon et al. 2010) based on a multiple alignment of 54 plant RPOT sequences (listed in Supplementary Table 2) with 100 bootstrap replicates. Prefixes of designations of plant RpoT proteins refer to the abbreviations used in Supplementary Table 2. The tree was rooted with four *green* algae proteins. *Asterisks* indicate that experimental evidence for the localization in mitochondria, plastids or both organelles exists. Otherwise, the indicated localization (RPOTm-mitochondrial enzyme, p-plastid enzyme, and mp-enzyme targeted to both mitochondria and plastids) is solely based on the position in the tree. Based on the experimental data shown in Fig. 4, *PpRpoT3*/PpRPOT3 was renamed into *PpRpoTm*/PpRPOTm

No conclusive evidence concerning the phylogenetic relationship of the three *Physcomitrella RpoT* genes can be inferred from the exon–intron structure. While the acquisition of intron 1 (as of *PpRpoTmp1*) is in favour of a closer relationship of *PpRpoTmp1* and *PpRpoTmp2*, *PpRpoTmp1* and *PpRpoT3* both share the lack of the highly conserved intron 16 (as of *Arabidopsis RpoTm*). To assess the phylogenetic positioning of the polymerase characterised in the present study more specifically, phylogenetic trees were reconstructed applying the Bayesian algorithm as well as maximum-likelihood (PhyML) analysis. Tree reconstruction was based on a multiple alignment of 54 plant RPOT sequences (see Fig. 3; Supplementary Table 2). Bayesian as well as PhyML analysis resulted in essentially the same tree topology (not shown). Figure 3 shows the rooted (green algae) consensus tree of the PhyML analysis in which angiosperm RPOT polymerases constitute two clearly discernible groups: one consisting of plastid-localised polymerases, and the other of mitochondrial-localised and dual-targeted enzymes, while *Selaginella* and *Physcomitrella* polymerases do not belong to these clusters. All *Physcomitrella* RPOTs appear to be the result of two *Physcomitrella* lineage specific duplication events, with the *PpRpoTm/RpoTmp1* duplication as the more recent one. Furthermore, conservation of the plastid-targeting properties for both dual-targeted proteins or, alternatively, independent acquisition of plastid-targeting properties for both RPOTmp1 and RPOTmp2 is supportive for their functional importance in *Physcomitrella*. The lack of a dual-targeted RPOTmp both in *Nuphar* and in monocots supports the view that the RPOTmp enzyme detected in eudicots is an evolutionary novelty due to an *RpoTm* gene duplication that likely occurred only after the separation of monocots and eudicots. The putative plastid-targeting sequences as present in two of the three *Physcomitrella* RPOT proteins are, therefore, clearly species- or lineage-specific convergent inventions. Importantly, multiple mitochondrial RNA polymerases as found in *Physcomitrella* and eudicots are identified in *Nuphar* as well. The fixation of duplicated *RpoT* genes led to a convergent multiplicity of mitochondrial RNAPs in *Nuphar*, *Physcomitrella* and eudicots, previously not found in any other eukaryotic lineage.

### Mitochondrial localisation of the phage-type RNA polymerase

**Fig. 4:**
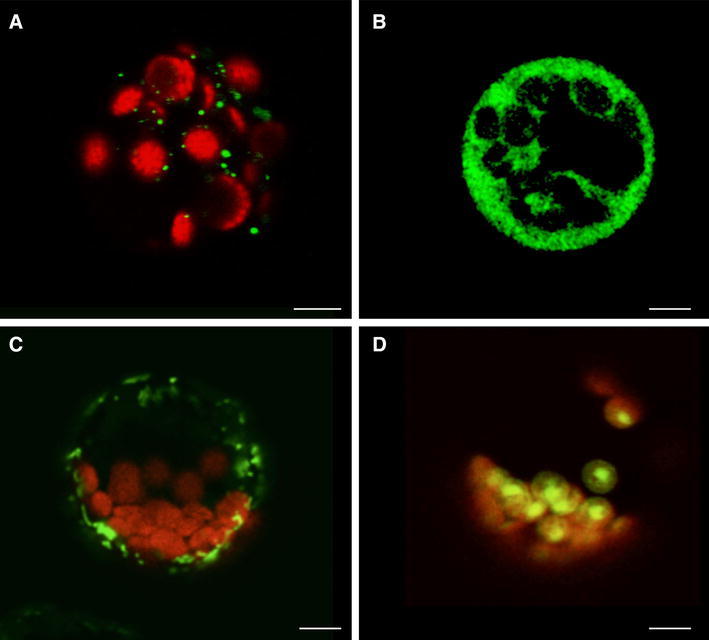
Transient expression of GFP fusion proteins in *A. thaliana* protoplasts. The *PpRpoT3* gene fragment encoding a putative transit peptide was inserted into plasmid pOL-GFPS65C to generate a vector driving the expression of utrPpRpoT3 (**a)** showing mitochondrial GFP localisation; RpoT3 was therefore designated PpRpoTm. Control constructs code for GFP (**b**, pOL only), mitochondrial CoxIV-GFP (**c**) and plastidial RecA-GFP (**d**), respectively. Images were taken by confocal fluorescence microscopy. *Scale bar* 10 μm

While both *PpRpoTmp1* and *PpRpoTmp2* contain two in-frame AUG codons in their 5′ ends, potentially facilitating dual targeting properties due to alternative translational starts, for *PpRpoT3* only one putative start codon was identified. Computer analysis of the putative transit peptide (Emanuelsson et al. 2000) produced high scores (0.8–0.9) for mitochondrial localisation and no indication for targeting to chloroplasts. To experimentally investigate the subcellular localisation of the *PpRpoT3*-encoded polypeptide, the putative N-terminal transit peptide was fused in-frame to the GFP coding region. Furthermore, an additional construct was generated in such a way that it contained the whole native 5′UTR sequence together with the sequence encoding the putative transit peptide, allowing for translational initiation at potential non-AUG start codons and to rule out GFP localisation due to forced translational initiation (Kabeya and Sato 2005). After transfection of the *PpRpoT3*-GFP fusion constructs into *Arabidopsis* protoplasts transient expression was monitored by confocal laser scanning microscopy. Three plasmids encoding GFP only (Fig. 4b), a mitochondrial Cox4-GFP and a plastid RecA-GFP (Fig. 4d) fusion protein were used for reference transformations of *Arabidopsis* protoplasts. Protoplasts expressing PpRPOT3-GFP from both constructs displayed green fluorescence of small structures (shown in Fig. 3a for the construct with 5′UTR) resembling the fluorescent mitochondria of protoplasts synthesizing Cox4-GFP (Fig. 4c), substantiating a mitochondrial localisation of PpRPOT3. The obtained data indicates that the N-terminal part of PpRPOT3 has mitochondrial targeting properties and strongly suggests that RPOT3 is exclusively targeted to mitochondria *in planta.* Therefore, PpRPOT3 will be subsequently designated as PpRPOTm.

**Fig. 5:**
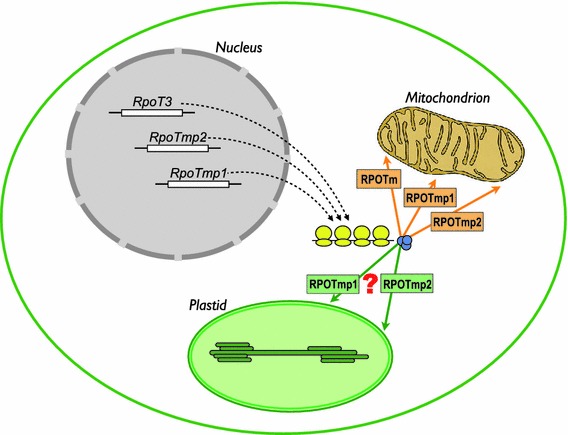
Proposed localisation of the three phage-type RNA polymerases in *Physcomitrella* cells. Based on the results of the present study. RPOT3 is localised exclusively to mitochondria and was, therefore, renamed into RPOTm. RPOTmp1 and RPOTmp2 are potentially targeted to both mitochondria and plastids; their localisation might be regulated via usage of different start codons during translation and vary between different tissues

In the light of the existence of only one *RpoT* gene in *Selaginella*, the finding of three genes encoding organellar phage-type RNA polymerases in the nuclear genome of *P. patens* comes as a surprise (Fig. 5). It raises the question as to the functions of three RNA polymerases (PpRPOTm, PpRPOTmp1, PpRPOTmp2) in mitochondria and of possibly also three enzymes in chloroplasts (PEP and the putative NEP activities PpRPOTmp1 and PpRPOTmp2 in addition to PEP). Multiple RPOT activities per organelle appear as a common feature of eudicotyledonous plants and *Physcomitrella*. Whether this complexity is due to an evolutionary trend, adaptive or neutral, remains to be shown. The identification of different but overlapping functions of RPOT proteins in plastids as well as in mitochondria of *Arabidopsis* is only the first step to answer such a question, since a regulatory role sensu stricto for these enzymes remains to be shown (Liere et al. 2011). *Physcomitrella* *RpoT* deletion mutants and *Physcomitrella* lines with manipulated higher or lower RPOT activities might offer a way to show if a similar division of labour evolved in case of the three mitochondrial RNA polymerases in *Physcomitrella* as compared to the two *Arabidopsis* enzymes (Kühn et al. 2009).

## Electronic supplementary material

Below is the link to the electronic supplementary material.

Supplementary material 1 (PDF 108 kb)
