# Supplementary material for: A third mitochondrial RNA polymerase in the moss Physcomitrella patens
Source: Curr Genet. 2013 Sep 12;60(1):25–34. doi: 10.1007/s00294-013-0405-y (PMC3895441; doi:10.1007/s00294-013-0405-y)
Supplement: Supplementary file 1 — Supplementary material 1 (PDF 108 kb) [file 294_2013_405_MOESM1_ESM.pdf]

Current Genetics

A third mitochondrial RNA polymerase in the moss *Physcomitrella patens*

Uwe Richter[1,2], Björn Richter[1], Andreas Weihe[1], Thomas Börner[1,3]

Institut für Biologie - Genetik, Humboldt-Universität zu Berlin, Chausseestr. 117, 10115 Berlin, Germany; [2]present address: Research Programs Unit, Molecular Neurology, University of Helsinki, Haartmaninkatu 8, 00290 Helsinki, Finland; [3]correspondence to: thomas.boerner@rz.hu-berlin.de

[3]Corresponding author: e-mail: thomas.boerner@rz.hu-berlin.de, phone: +493020938140, fax: +493020938141

Supplementary Table 1: Oligonucleotides for *PpRpoT3* cDNA cloning

Oligo Name	Oligo Sequence (5‘ – 3‘)
5racePpT3gsp1	CCAACTGAGACGGAGGCAAC
5racePpT3gsp2	GCACTACGGTAAGGATTCAAAGACC
5racePpT3gsp3	AGTTTGCACTTCCGCTGTCG
5racePpT3gsp4	AACTTGCGCAATCCTTGACC
5racePpT3gsp5	GACCTGCTGCCTCCACAATG
5racePpT3gsp6	CCTCCACAATGCAACAATTCG
5racePpT3gsp7	AAGCAGAAATAAGAGAGAGAGGGAGC
3racePpT3gsp1	GGAGTCCTGGACATCAGGGAGG
3racePpT3gsp2	TGGACATCAGGGAGGTTCTTAAAGCCC
3racePpT3gsp3	TGCTCTCTTTGCAACTGGGTGACG
3racePpT3gsp4	TTCAGACCAACGTCGGAATGG
PpT3p43	CATTGTGGAGGCAGCAGGTC
PpT3m3169	TCCATTCGTCACCCAGTTGC
Seq4T3fw	ACGAGTTCCGAGCGACGATCC
Seq3T3rev	ACCAAAGGATTGCACTCCACAACACC
Seq2T3fw	TGCAACATTATGCAGCCCTTGG
Seq1T3rev	CGCTGCATGTTTACTGGCCTCGG
PpT3exon2	GCCTTTCCTCTTCGTTTCGT

Supplementary Table 2. List of RpoT sequences used for phylogenetic tree construction

Abbr.	Taxon/Name/Homolog	Annotation
AlRpoTm	Arabidopsis lyrata RpoTm	EFH64954
AlRpoTmp	Arabidopsis lyrata RpoTmp	EFH49996
AlRpoTp	Arabidopsis lyrata RpoTp	EFH56807
AtRpoTm	Arabidopsis thaliana RpoTm	P92969
AtRpoTmp	Arabidopsis thaliana RpoTmp	CAC17120
AtRpoTp	Arabidopsis thaliana RpoTp	O24600
BdRpoTm	Brachypodium distachyon RpoTm	Bradi4g08880 (http://www.phytozome.com)
BdRpoTp	Brachypodium distachyon RpoTp	Bradi1g31070 (http://www.phytozome.com)
BoRpoTm	Brassica oleracea RpoTm	XP_002308414.1
CaRpoTm	Chenopodium album RpoTm	CAA69305
CsRpoTmp	Cleome spinosa RpoTmp	DQ415921
CusRpoTm	Cucumis sativus RpoTm	Cucsa.284310 (http://www.phytozome.com)
CusRpoTmp	Cucumis sativus RpoTmp	Cucsa.238450 (http://www.phytozome.com)
CusRpoTp	Cucumis sativus RpoTp	Cucsa.090030 (http://www.phytozome.com)
HvRpoTm	Hordeum vulgare RpoTm	AJ586899
HvRpoTp	Hordeum vulgare RpoTp	AJ507396
MgRpoTm	Mimulus guttatus RpoTm	mgv1a000798m (http://www.phytozome.com)
MgRpoTmp	Mimulus guttatus RpoTmp	mgv1a000681m (http://www.phytozome.com)
MgRpoTp	Mimulus guttatus RpoTp	mgv1a000925m (http://www.phytozome.com)
MipuRpoT	Micromonas pusilla RpoT	EEH56417.1
MspecRpoT	Micromonas spec RpoT	XP_002503703.1
NaRpoTm1	Nuphar advena RpoTm1	FN811768
NaRpoTm2	Nuphar advena RpoTm2	FN820498
NaRpoTp	Nuphar advena RpoTp	FN811769
NsRpoTm	Nicotiana sylvestris RpoTm	AJ416568
NsRpoTmp	Nicotiana sylvestris RpoTmp	AJ302019
NsRpoTp	Nicotiana sylvestris RpoTp	AJ302020
OlRpoT	Ostreococcus lucimarinus RpoT	ABO98141
OsRpoTm	Oryza sativum RpoTm	AB096014
OsRpoTp	Oryza sativum RpoTp	AB096015
OtRpoT	Ostreococcus tauri RpoT	CAL55557.1
PotRpoTm1	Populus trichocarpa RpoTm1	EEE88785
PotRpoTm2	Populus trichocarpa RpoTm2	EEF02167
PotRpoTmp	Populus trichocarpa RpoTmp	EEF10826
PotRpoTp1	Populus trichocarpa RpoTp1	EEF03684
PotRpoTp2	Populus trichocarpa RpoTp2	EEE91937
PpRpoTm	Physcomitrella patens RpoTm	in this work
PpRpoTmp1	Physcomitrella patens RpoTmp1	CAC95163
PpRpoTmp2	Physcomitrella patens RpoTmp2	CAC95164
RcRpoTm	Ricinus communis RpoTm	EEF47677
RcRpoTmp	Ricinus communis RpoTmp	EEF28786
RcRpoTp	Ricinus communis RpoTp	EEF33998
SbRpoT1	Sorghum bicolor RpoT1	XM_002460990
SbRpoTp	Sorghum bicolor RpoTp	XM_002437329
SmRpoTm	Selaginella moellendorfii RpoTm	CAP70041
SoRpoTmp	Spinacia oleracea RpoTmp	Y18852
SoRpoTp	Spinacia oleracea RpoTp	Y18853
TaRpoTm	Triticum avestivum RpoTm	AAF32492
TaRpoTp	Triticum avestivum RpoTp	AAB01085
VvRpoTm	Vitis vinifera RpoTm	AM483136
VvRpoTmp	Vitis vinifera RpoTmp	AM488491
VvRpoTp	Vitis vinifera RpoTp	AM453066
ZmRpoTm	Zea mays RpoTm	AAD22977
ZmRpoTp	Zea mays RpoTp	AAD22976
